# QuantISH: RNA in situ hybridization image analysis framework for quantifying cell type-specific target RNA expression and variability

**DOI:** 10.1038/s41374-022-00743-5

**Published:** 2022-02-15

**Authors:** Sanaz Jamalzadeh, Antti Häkkinen, Noora Andersson, Kaisa Huhtinen, Anna Laury, Sakari Hietanen, Johanna Hynninen, Jaana Oikkonen, Olli Carpén, Anni Virtanen, Sampsa Hautaniemi

**Affiliations:** 1 Research Program in Systems Oncology, Research Programs Unit, Faculty of Medicine, University of Helsinki, Helsinki, Finland; 2 Institute of Biomedicine and FICAN West Cancer Centre, University of Turku and Turku University Hospital, Turku, Finland; 3 Department of Obstetrics and Gynecology, University of Turku and Turku University Hospital, Turku, Finland; 4 Department of Pathology, University of Helsinki and HUS Diagnostic Center, Helsinki University Hospital, Helsinki, Finland

**Keywords:** Cancer imaging, Transcriptomics

## Abstract

RNA in situ hybridization (RNA-ISH) is a powerful spatial transcriptomics technology to characterize target RNA abundance and localization in individual cells. This allows analysis of tumor heterogeneity and expression localization, which are not readily obtainable through transcriptomic data analysis. RNA-ISH experiments produce large amounts of data and there is a need for automated analysis methods. Here we present QuantISH, a comprehensive open-source RNA-ISH image analysis pipeline that quantifies marker expressions in individual carcinoma, immune, and stromal cells on chromogenic or fluorescent in situ hybridization images. QuantISH is designed to be modular and can be adapted to various image and sample types and staining protocols. We show that in chromogenic RNA in situ hybridization images of high-grade serous carcinoma (HGSC) QuantISH cancer cell classification has high precision, and signal expression quantification is in line with visual assessment. We further demonstrate the power of QuantISH by showing that *CCNE1* average expression and *DDIT3* expression variability, as captured by the variability factor developed herein, act as candidate biomarkers in HGSC. Altogether, our results demonstrate that QuantISH can quantify RNA expression levels and their variability in carcinoma cells, and thus paves the way to utilize RNA-ISH technology.

## Introduction

RNA in situ hybridization (RNA-ISH) spatial transcriptomics technology allows identification of gene expression profiles in individual cells while preserving information of the spatial tissue context^1,2^. The ability to analyze expression localization and tumor heterogeneity in tissue sections has opened new avenues for cancer research^3–12^. As RNA expression levels are functional and reflect both genetic and epigenetic aberrations, transcript-based biomarkers are paving their way also to the clinical setting, when protein expression is not specific enough or the relevant protein to be targeted is not known. For instance, a recently introduced single-molecule non-radioisotopic RNA-ISH technology RNAScope^3^ is particularly useful both in molecular pathology for clinical diagnostics and in research settings utilizing formalin-fixed, paraffin embedded (FFPE) samples that often contain partially degraded RNA^5,6^.

Computational approaches for RNA signal quantification and spatial expression analysis have several potential advantages to visual quantification, such as increased time efficiency and higher reproducibility. Open-source tools, such as smiFISH and FISH-quant^13^, are available for fluorescent unamplified single molecule in situ hybridization target RNA dot detection. To our knowledge, two open-source methods (SMART-Q and dotdotdot^14,15^) are available for quantifying fluorescent amplification-based RNAScope in situ hybridization signal. However, these enjoy the benefits of multiple separate channels for RNA labeling and nuclear counterstaining, and utilize cell type specific markers for cell classification purposes.

Staining with chromogenic labeling is easier and more feasible to incorporate to routine large-scale production in pathology laboratories in terms of existing equipment and archival properties^16^. In comparison to fluorescent RNA in situ hybridization (RNA-FISH), images from RNA-ISH experiments with chromogenic labels (chromogenic in situ hybridization; RNA-CISH), require more a sophisticated, discriminatory analysis both for nuclear segmentation and marker quantification, as the two features are superimposed on a single channel. Further, chromogenic immunohistochemistry (IHC) image analysis methods^17–20^, are not directly useful because single-molecule RNA markers manifest as individual or clustered dots present both in the nucleus and the cytoplasm of cells, unlike tagged proteins. While RNA-CISH images are more difficult to analyze than RNA-FISH or IHC, the recognizable counterstained cell structures in the background of superimposed signal dots allow for morphology-based identification of different cell types without separate cell type markers.

Here, we present QuantISH, an open-source, versatile image analysis pipeline capable of cell type-specific expression quantification of RNA-CISH signals in carcinoma, stromal, and immune cells in digitalized singleplex images. QuantISH is designed to identify individual cell types based on their nuclear morphology, and quantify expression signal at the level of individual cells. QuantISH has a modular design, which ensures that QuantISH is applicable to wide range of image analyses with minor modifications to allow adaptation to different image and sample types and staining protocols. Modularity also allows straightforward change of the methods to more powerful ones when such come available. We also introduce herein a variability factor that takes advantage of the RNA-ISH spatial transcriptomics technology by characterizing the biological variability of a gene expression in a sample independently of the variation exerted by the mean expression, which allows quantitative comparison of expression heterogeneity between samples.

To demonstrate the utility of QuantISH, we analyzed chromogenic RNA-ISH images from patients with high-grade serous carcinoma (HGSC), which is the most common tubo-ovarian cancer subtype with only 39% five-year survival rate^21^. HGSC is copy-number driven cancer in which *CCNE1* amplification frequency is 15–20%^22^. While *CCNE1* amplifications are predictive for HGSC five-year survival, especially when combined to protein overexpression^23,24^, evidence of survival association is sparser for *CCNE1* gene expression^25^. Here, we quantified whether the cancer cell specific gene expression levels and variability of *CCNE1* and *DDIT3*, a proapoptotic transcription factor found to be part of the stress-associated tumor cell population enriched after chemotherapy in HGSC^26^, are associated with patient outcomes.

## Methods

### Patient characteristics

Two cohorts were used in the analysis. The Helsinki cohort is a retrospective cohort, and the HERCULES/DECIDER cohort a prospective one.

The Helsinki cohort includes patients treated at Helsinki University Hospital for high-grade serous carcinoma. Samples were obtained from Helsinki biobank. A subcohort of 95 patients was selected for this study based on the following criteria: tubo-ovarial origin, stage III-IV at diagnosis, primary debulking surgery, and at least six cycles of adjuvant platinum-based chemotherapy. The median age was 63 years (min: 40, max: 82) at diagnosis. Surgery outcome was R0 (no residual tumor) for 21 patients and R > 0 (residual tumor after surgery) for 74 patients^27^. The patients’ median progression free interval (PFI) from last treatment administration until disease progression or end of follow up was 10 months (min: 0, max: 165) and the median overall survival time from diagnosis until last follow up or death was 54 months (min: 10, max: 170). The histological diagnosis was re-evaluated and confirmed according to current diagnostic criteria^28^. Tissue material used was collected into tissue microarray blocks (TMAs) that for each patient included, on average, six one millimeter cores: 3 cores from adnexal (ovary, fallopian tube) tumor and 3 cores from omental/peritoneal tumor comprising altogether 556 tumor samples.

The prospective HERCULES/DECIDER cohort consists of primary debulking surgery (PDS) and neoadjuvant chemotherapy (NACT) treated HGSC patients with complete clinical and follow-up data. The subcohort used in this project consists of 10 NACT patients treated at Turku University Central Hospital. NACT consisted of median three (range 3-4) cycles of carboplatin and paclitaxel chemotherapy. For those 9 patients who responded to NACT, an interval debulking surgery (IDS) was performed, aiming for optimal cytoreduction, followed by a median of three (range 2–6) cycles of adjuvant chemotherapy. Tissue material used for this cohort came from diagnostic (chemotherapy naïve) FFPE blocks of ten patients.

### RNA in situ hybridization and imaging for Chromogenic RNA-ISH

The set of five tissue microarray slides from the Helsinki cohort were stained with chromogenic RNA-ISH (RNA-CISH) for *CCNE1* and *DDIT3*, as well as for *PPIB* to act as a positive control and evaluate the RNA quality in the FFPE tissue. Detailed hybridization protocols and imagining information can be found in the Supplementary material.

### Tissue microarray RNA-CISH image pre-processing

#### Slide scanner data extraction

The TMA scans were received in MIRAX (MRXS) format files, containing a hierarchical pyramid of the scanned images and metadata, from a 3DHISTECH Pannoramic 250 FLASH II digital slide scanner with 40x magnification^29^.

We extracted contiguous images from the tiled microscope scans and cropped the TMA spots using the full resolution layer of the scanned full slide images for the quantitative analysis using a customized QuantISH module. The process uses a linear transformation recorded by the slide scanner to align each captured frame into the final output image such that the overlapping areas have been removed (Supplementary Fig. 1A).

#### Cropping TMA spots

**Fig. 1:**
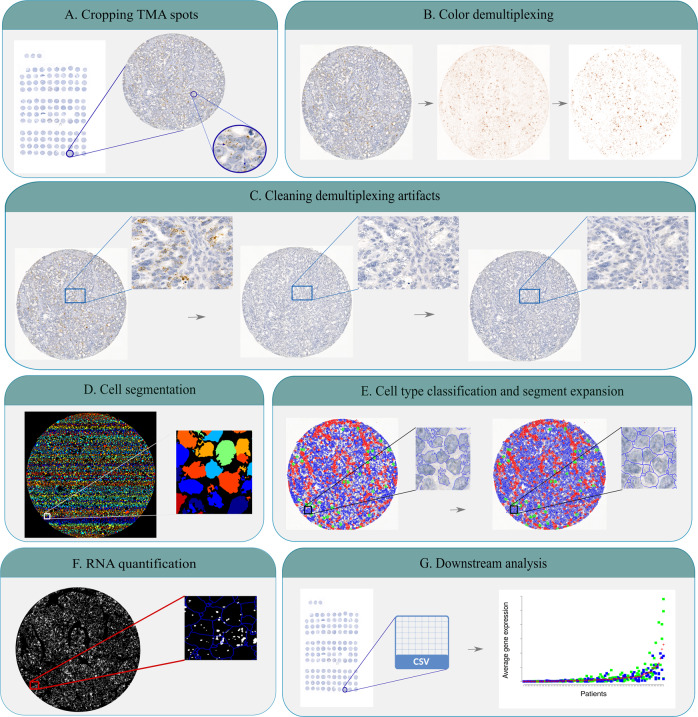
Overview of QuantISH image analysis pipeline. **A** Cropping chromogenic tissue microarray (TMA) spots. **B** Separating the brown chromogenic signals from the original TMA images using the color separation and background noise suppression steps. **C** Cleaning the original chromogenic image from the separated marker channel (brown) to preserve the cellular morphology under a high level of marker expression for further segmentation in the nuclear staining (blue). **D** Cell segmentation of a cleaned nuclear staining channel for a TMA image. Different colors represent different objects (cells). **E** Cell type classification and segment expansion of the nuclear segments to cover the cell cytoplasm. **F** Using the separated marker channel to quantify RNA signals inside each TMA spot and each specific cell type. **G** Downstream analysis of average expression and expression variability per sample/patient and their association to patients’ survival.

To extract the TMA spots from the whole slide image, we implemented a module based on the HistoCrop method^30^, to crop each TMA spot into a separate image file for downstream analyses (see Fig. 1A and Supplementary Fig. 1B for more details).

#### Color demultiplexing

Since the RNA-ISH stain and nuclear counterstain are superimposed in the RNA-CISH images, direct cell segmentation and cell type-specific classification would be imprecise in the presence of RNA markers. We separated brown marker RNA stain from blue nucleus stain in each TMA spot into separated channels for cell type classification and RNA quantification using color deconvolution plugin within the ImageJ software^31,32^. As the separated marker RNA signal channel obtained from the ImageJ plugin suffered from background noise, we applied a Renyi entropy thresholding method^33^ to filter out the background (Fig. 1B).

#### Cleaning demultiplexing artifacts

To mitigate the effect of voids in the demultiplexed nucleus staining due to overlapping signals in RNA-CISH images resynthesizer textural synthesis plug-in^34^ for GNU Image Manipulation Program (Gimp) software (version 2.8) was used. This allows the usage of standard cell segmentation algorithms. The plug-in is based on the algorithm presented in^35^, which performs best-fit texture synthesis on a user-specified region of interest in an image. The steps were implemented in Python-Fu scripting language, which allows automatic batch processing for each TMA spot.

The void-filled nucleus signal (Fig. 1C) and the demultiplexed marker RNA signal from the previous section were used in the subsequent analysis.

### Cell segmentation and classification in RNA-CISH images

#### Cell segmentation

We used CellProfiler software (version 3.1.8), to segment the filtered nucleus signals of each TMA spot into individual cells^36,37^. First, we used the RescaleIntensity component to scale the intensity into an appropriate range followed by IdentifyPrimaryObjects component for segmentation and Otsu’s method^38^ with adaptive thresholding. To separate clumped objects, we used object shape instead of intensity. The non-default parameters were determined experimentally using 20 sub-images and five iterations: object diameter 25 to 170 pixels, and threshold smoothing scale of 1.3488. Segmentation of a single TMA spot is shown in Fig. 1D.

#### Cell type classification

The segmented objects (nuclei) in each TMA spots were classified into cancer, immune, and stromal cells using the filtered nucleus channel and its segmentation. For this, we extracted the area, the mean nucleus stain intensity, the eccentricity, and the perimeter-to-area ratio of each segmented object. Supplementary Fig. 3A exemplifies the features used in training the classifier.

To classify the cells, we trained a supervised quadratic classifier using 360 cells with the four extracted features as an input. The outputs of the classifier were “cancer cell”, “immune cell”, “stromal cell”, and three artifact classes (“small”, “cell-sized irregular”, and “large”). The quadratic classifier was implemented in MATLAB and was trained with uniform class priors (Supplementary Fig. 3B).

#### Evaluation of cancer cell classification accuracy

Since we were primarily interested in cancer cell specific expression, the precision of cancer cell classification performance was calculated against the annotations by a pathologist in an unblinded way. Six different RNA-CISH TMA spots were selected to represent different types of tissue composition in ovarian and omental tumor, and the classification results containing the different cell types were given to pathologists for annotation.

#### Improving classification performance using spatial information

Cells tend to be spatially co-localized by the cell type in the tissue, implying that classification benefits by labeling neighboring cells with the equal classes. We extracted spatial probability maps for each cell type from the quadratic classifier, which were then low-pass filtered in logarithmic space (probability product space) using a disk kernel of 100 pixel radius (cf. cell radius of ~25). This propagates the probability of classification to neighboring cells in the regions with large classification uncertainty, but allows a cell exhibiting strong features of a particular type to retain its class.

#### Segment expansion

To include cytoplasmic RNA signals, we expanded the segments to include the cellular cytoplasm. This expansion was done by dilating the segments in the unlabeled space with a disk kernel with radius of size 20, 5, and 5 pixels for the cancer, immune, and stromal classes, respectively. Ties were broken to the nearest segment, to not expand the cells over their neighbors. The parameters were tuned experimentally to account for the differences in the morphology of the different cell types. Figure 1E shows an example of the cell classification and segment expansion for one TMA spot.

### RNA signal quantification in RNA-CISH images

Before marker RNA quantification, we removed nonspecific signals that manifest as intensively stained regions in the isolated RNA channel. We masked the extreme regions up to 5/256 (~ 2%) bins from total black color in the marker signal image and expanded the regions up to 10 pixels using a disk kernel (Fig. 1F). After this, the RNA signal intensities for *CCNE1*, *DDIT3* and *PPIB* were integrated (summed) inside each individual cell in three separate image sets, and a list of all the cell classes, IDs linking them to their location on the slide, and the corresponding marker RNA intensities were obtained for each TMA spot. Figure 1G highlights the RNA quantification inside each individual TMA spot.

As QuantISH does not perform absolute RNA level quantification, the reported expression values are in intensity units. Considering that the intensity of each image of a TMA spot is interpreted in the scale of [0, 1], the average expression quantification value is only “1” when a cell is fully saturated with the chromogenic signals. For a typical image, the intensity is very much below the saturation point, and most of the cellular area is not covered by the dots. Thus, the average intensity values typically are low, such as from 0.001 to 0.01. Of note, the average expression values are often experiment specific because the intensity levels may be highly dependent on the staining protocol, etc.

*PPIB* was used as a positive control gene to evaluate RNA quality in the same tissues that were subject to the analysis of specific RNA expression (*CCNE1* or *DDIT3*). At least moderate level *PPIB* expression was required for reliable analysis of the other genes. For this purpose, Otsu’s thresholding was used for a two-class classification (negative and positive) of *PPIB* intensities to filter out the unreliable spots. The method optimizes a threshold to separate the low and high intensity groups by maximizing inter-class variance (which minimizes intra-class variance) (Supplementary Fig. 4). After excluding the cores with less than moderate expression, expression analysis for *CCNE1* and *DDIT3* in the Helsinki cohort consisted of 354 TMA spots from 92 HGSC patients.

### Statistical analysis

#### Statistical analysis of expression values

We performed variance analysis (ANOVA) on cancer cell specific intensities to quantify the between patient variability, between phase (primary/metastases) variability, and the between spot variability. The ANOVA was implemented using a custom QuantISH module, performing ANOVA on nested factorial linear models, which can be computed in linear instead of the cubic time of a general model.

#### Variability analysis

**Fig. 2:**
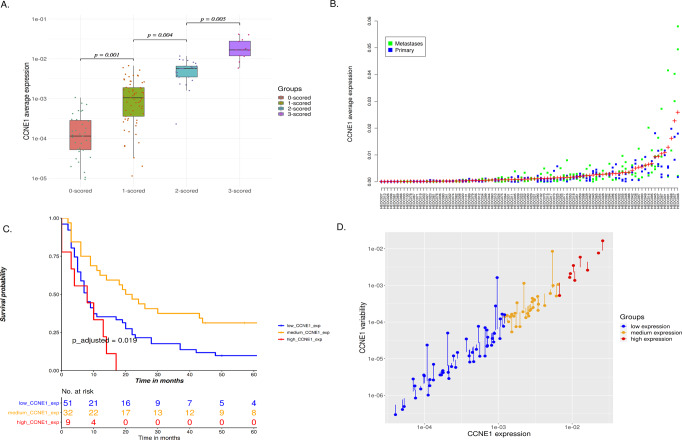
Association between *CCNE1* expression and high grade serous ovarian cancer patient survival. **A** Concordance between *CCNE1* cancer cell expression quantified by QuantISH and visual evaluation for 192 spots. In visual evaluation expression was scored from 0 (weak signal) to 3 (strong signal). The dots represent individual samples, lines medians, the boxes 25–75%, and the brackets indicate the significance of the difference between the QuantISH’s quantified expression between the four score groups. **B** *CCNE1* cancer cell specific expression in the 92 HGSC patients of our cohort. The dots in the plot represent average cancer cell expression in the multiple TMA spots for each patient and the plus sign shows average expression per patient. **C** For survival analysis, the samples are divided into three groups of low (*n* = 51), intermediate (*n* = 32), or high average expression (*n* = 9) patients. Progression free interval of the three groups of patients with low, intermediate, or high *CCNE1* average expression representing significant difference between three groups of patients. **D** *CCNE1* variability inside the three groups of patients with low, intermediate, or high *CCNE1* average expression. The stems represent the logarithmic variability factors. Expression is shown in intensity units, as described in the methods section.

In addition to the average expression, QuantISH captures the expression variability both inside *(i.e*., between individual cells) and between each individual spot (representing different tumor areas) from a single patient. We computed the weighted mean and variance of mean cell intensity, including the cell area as the weight, to give more weight to the larger cancer cells, in which the average can be more reliably quantified, and less weight to the small ones, essentially weighing out any small artifacts that could remain after the classifier. The weighted mean expression and weighted expression variance for each TMA spot/patient are computed as follows:

$$m = \mathop {\sum }\limits_i \left( {\frac{{Area_i}}{{\mathop {\sum }\nolimits_i Area_i}}} \right) \ast \left( {\frac{{Intensity_i}}{{Area_i}}} \right) = \left( {\frac{{\mathop {\sum }\nolimits_i Intensity_i}}{{\mathop {\sum }\nolimits_i Area_i}}} \right)$$

$$v = \mathop {\sum }\limits_i \left( {\frac{{Area_i}}{{\mathop {\sum }\nolimits_i Area_i}}} \right) \ast \left( {\frac{{Intensity_i}}{{Area_i}} - m} \right)^2,$$

where *m* is the mean expression in each spot/patient. *Area*_*i*_ is the area of each individual cell, and the *Intensity*_*i*_ represents the total (sum) intensity inside each cell. As the mean and variance of expression are naturally correlated, we computed a variability factor to regress out the mean effect from the raw variance using regression model in logarithmic space (see Supplementary material). Variability factors are shown as stems from regression line in Fig. 2D. This is essentially a more general form of the Fano factor, i.e., the variance-to-mean ratio with a non-unit power law relationship, which is apparent in the data (Fig. 2D). Hence, the variability factor value captures the variance independently of the mean expression.

#### Survival analysis

Kaplan–Meier (K–M) curves were generated for survival analysis based on progression-free interval (PFI) and overall survival (OS). PFI is defined as the time from last cycle of platinum treatment to the cancer progression or end of follow-up, whereas OS is the time from diagnosis to death or end of the follow-up. The log-rank test was used to test the significance of the PFI/OS-based survival differences.

To group the patients in *CCNE1* expression survival analysis, we optimized the two thresholds of increasing average expression for a three-way grouping. This resulted in the three expression groups marked with different colors in Fig. 2B. More precisely, the optimization was done via 20 different combinations of thresholds resulting in the first 55% of patients as low expression group, and 36% vs. 9% of the remaining patients in the medium and high *CCNE1* expression groups, respectively. To explicitly control for the threshold optimization’s impact to statistical significance, we adjusted the nominal p-values with the Benjamini-Hochberg procedure^39^. In the case of two group *CCNE1* expression survival analysis, the median was used as the threshold. For the *DDIT3* survival analysis, both for average expression and the expression variability, we used the median as the threshold.

### Analysis of whole slide fluorescent RNA in situ hybridization images

To assess QuantISH’s ability to analyze fluorescent RNA in situ hybridization images and to validate the *DDIT3* expression variability as a biomarker in HGSC, whole tissue slides from the HERCULES/DECIDER cohort were stained with fluorescent RNA-ISH (RNA-FISH) for *DDIT3* gene. Detailed hybridization protocols, imagining information and quantitative analysis of the images are described in Supplementary material.

### Quantification performance evaluation

To compare the core specific overall RNA-CISH cancer signal expression level as determined by QuantISH and by manual (visual) scoring, we selected 35 patients with varying degree of *CCNE1* RNA expression in 192 TMA spots to be evaluated by a pathologist and a cell biologist, blind to the QuantISH results and clinical data. Digital images of the TMA slides were visually assessed and the average number of probe signals per tumor cell was determined for the entire tissue core using a slightly modified version of the manufacturers scoring system. The scores ranged between 0 and 3 (“0”: Negative or an average of less than 1 signal per tumor cell; “1”: 1–3 probes per tumor cell; “2”: 4–10 probes per tumor cell; “3”: >10 probes per cell). The four groups were then compared to the estimated expression in the *CCNE1* images. ANOVA was used to test the statistical significance of the differences between groups.

## Results

### Overview of the QuantISH analysis pipeline

QuantISH is a comprehensive RNA in situ hybridization (RNA-ISH) image analysis pipeline to quantify gene expression and variability in individual cells from images with chromogenic labeling (Fig. 1). QuantISH is designed based on modular software engineering principles^40^ and its main modules are: cropping TMA spots; color demultiplexing; cleaning demultiplexing artifacts; cell segmentation; cell type classification and segment expansion, followed by cell-to-cell quantification of the marker RNA abundance. The modules are coupled into a pipeline and can be customized by the analysis needs: whole-slide image analyses can omit the TMA spot segmentation module, multichannel imaging the color demultiplexing (Supplementary Fig. 5), and the cell segmentation and classification modules can be easily customized for different staining protocols and tissue content by training the classifier with a modest number (less than 100) of cells per type. Moreover, the analysis allows quantifying both the average expression level and the variability within the tissue samples.

### Performance of cancer cell classification in comparison to visual assessment

Precision of cancer cell classification was computed as the proportion of correct calls in all expert annotated cancer cell calls, *i.e*., Precision = *TP*/(*TP* + *FP*), where *TP* is true positive and *FP* false positive. Overall, we found that the QuantISH cancer cell classification performs well with an overall precision of 0.88 (range 0.7–0.98) for the six individual spots with varying tissue composition, containing 16 218 annotated cancer cells.

### Accuracy of gene expression estimation in comparison to visual scoring

We compared the concordance of QuantISH quantified expression values for *CCNE1* to RNA-ISH visual scoring signal expression. The distribution of the QuantISH quantified expression values correlate well with visual scoring (*p* = 1.9 ×10^−9^; ANOVA) and differentiate between any two scoring bins (*p* < 0.005; ANOVA), as shown in Fig. 2A.

### *CCNE1* expression values predict ovarian cancer patient survival

To explore the impact of tissue site to *CCNE1* expression, we compared the differences in the *CCNE1* expression averaged over all the patients across all tissue sites (tubo-ovarian, omental, mesenterial, peritoneal) in the Helsinki TMA cohort. The *CCNE1* expression differences between tissue sites were not found to be significant (*p* = 0.78; ANOVA), which suggests that on average, tissue site plays a minor role in *CCNE1* expression, and survival analysis could be done without tissue site stratification.

**Fig. 3:**
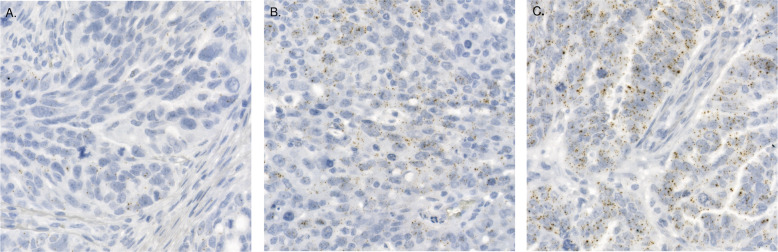
Examples of three *CCNE1* average expression predictive groups. **A** Sub-image of one TMA spot of patient HEOC036 containing a low level of RNA marker for *CCNE1* inside the cancer cells. **B** A medium level of *CCNE1* marker inside the cancer cells shown in a sub-image of one TMA spot of patient HEOC032. **C** Sub-image of one TMA spot of patient HEOC087 containing a high level of RNA marker signal for cancer cell *CCNE1* expression.

We then proceeded to test whether *CCNE1* RNA expression is associated with survival. Dividing the *CCNE1* expression to two groups based on median expression value did not result in significant survival association (*p* = 0.81). Hence, following the findings by Etemadmoghadam et al.^41^, we divided the patients into three groups based on their *CCNE1* expression values and used platinum free interval (PFI) and overall survival (OS) as the outcomes in a survival analysis. We optimized the thresholds using sorted average expression values resulting in 55% of the patients as low *CCNE1* expression group, and 36% *vs*. 9% of the remaining patients in the medium and high *CCNE1* expression groups, respectively, while correcting p-values for the false discovery rate (Methods). The three-tier *CCNE1* expression groups were found to have a significant association to both PFI (*p* = 0.02; adjusted log-rank test; Fig. 2C) and OS (*p* = 0.02). Two group comparison is informative in PFI when split unequally (low and medium *vs*. high *p* = 0.02) and also in OS (low and medium *vs*. high *p* = 0.04). However, the low *vs*. medium difference is also significantly informative in PFI (*p* = 0.009) and OS (*p* = 0.035). Figure 3 represents three examples of tissues containing low, medium, and high level of cancer cell *CCNE1* expression. Association of *CCNE1* expression with PFI remained significant in patients without residual tumor after surgery (R0, *n* = 19, *p* = 0.01), but exhibited a non-significant but similar trend in patients with residual tumor (R > 0, *n* = 72, *p* = 0.08).

### Gene expression variability as a biomarker

We tested whether *CCNE1* or *DDIT3* expression variability associates with patient survival. As mean and variance are statistics that naturally related to each other, we used variability factors (see Methods) that capture the variance independently of the mean expression. For *CCNE1* in the Helsinki cohort, the association between the variability factor and PFI (*p* = 0.38; log-rank test) and OS (*p* = 0.37; log-rank test) were not significant. Further, the variability factors, as represented by the stems from the regression line in Fig. 2D, do not significantly differ between the three groups (*p* = 0.9; ANOVA; Supplementary Fig. 7). This implies that *CCNE1* expression exhibits variability between the individual tumor cells, but the degree of variability remains similar at all levels of expression.

**Fig. 4:**
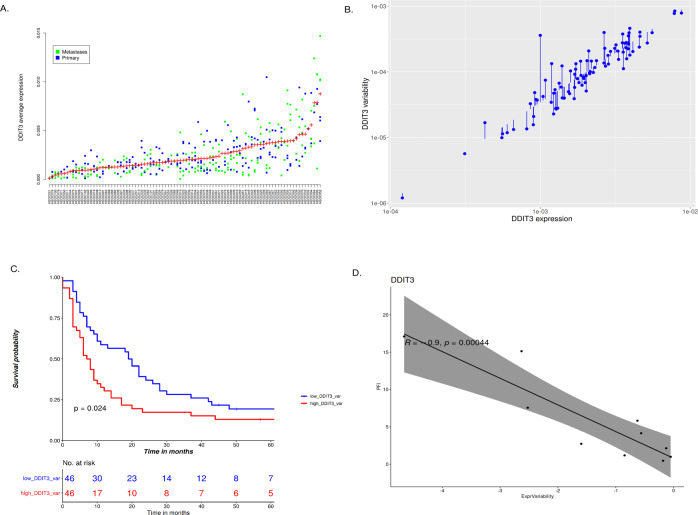
Capturing *DDIT3* expression variability and its association to patient survival. **A** *DDIT3* cancer cell expression across the 92 HGSC patients in the Helsinki cohort. The dots in the plot represent average cancer cell expression in the multiple TMA spots for each patient and the plus sign shows average expression per patient. **B** *DDIT3* variability across the 92 patients in the cohort. The stems represent the logarithmic variability factors. **C** Progression free interval of HGSC patients based on low and high 50% of *DDIT3* expression variability factor. **D** Significant negative correlation (R = −0.9, *p* = 0.0004) between the *DDIT3* gene expression variability and the progression free interval in the HERCULES/DECIDER cohort. The dots represent individual samples and the line the estimated trend with the gray area being its standard error. Expression is shown in intensity units, as described in the methods section.

**Fig. 5:**
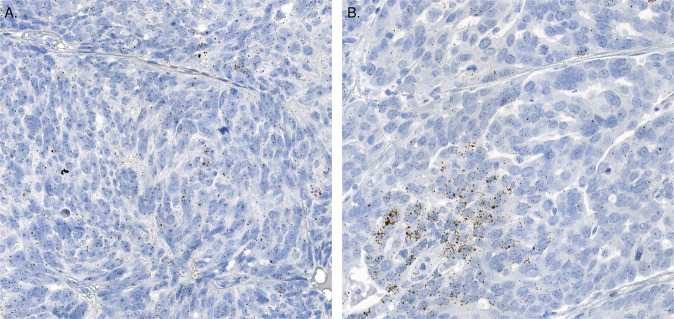
Example of two *DDIT3* expression variability predictive groups. **A** Sub-image of one TMA spot of patient HEOC056 representing a low level of RNA marker variability for *DDIT3* inside the cancer cells. **B** A high level of *DDIT3* expression variability inside the cancer cells shown in a sub-image of one TMA spot of patient HEOC0390.

For *DDIT3*, we analyzed both average expression values (Fig. 4A) and variability factors (Fig. 4B). First, we again confirmed that *DDIT3* expression is not tissue specific by testing for differences in the average expression values over all patients across all tissue sites (*p* = 0.72; ANOVA). Second, we found out that *DDIT3* average expression values do not have significant association with the PFI (*p* = 0.73; Supplementary Fig. 6) nor OS (*p* = 0.61). However, dividing the patients based on the variability factor resulted in a significant PFI association, as shown in Fig. 4C (*p* = 0.02). The same analysis for OS was not significant (*p* = 0.22), though the same trend is visible. The within-patient inter-core (between different tumor areas within one patient and tissue) expression difference for *DDIT3* is also apparent in Fig. 4A, unlike for *CCNE1* (cf. Fig. 2B). Figure 5 shows two examples of tissues representing low and high *DDIT3* expression variability in cancer cells respectively.

We further tested the *DDIT3* expression and variability factor survival association in the HERCULES/DECIDER cohort that contained IF-stained whole slide images from ten primary samples of ten patients. As expected, average *DDIT3* expression correlation with PFI was non-significant (*p* = 0.35), whereas the correlation between *DDIT3* expression variability and PFI was significant (*p* = 0.0004; Fig. 4D).

## Discussion

RNA in situ hybridization (RNA-ISH) is increasingly important and powerful spatial transcriptomics technology for cancer research and routine molecular pathology. Biomarker discovery and validation for diagnostic practices is time consuming and requires objective and consistent analysis of hundreds of samples. Gene expression patterns in tissues beyond mere average expression levels require robust and accurate computational methods. QuantISH is open-source software to answer these needs and its modular design allows its use for various applications as well as inclusion of new methods. Particularly, different segmentation algorithms can be easily substituted into the modular QuantISH pipeline, and the classification step is also easily trainable with new cell types annotated by user for any new set of RNA-CISH images or can also be replaced with alternative machine learning algorithms.

We showed that QuantISH overcomes the challenges of automatic quantification of chromogenic RNA in situ hybridization (RNA-CISH) images, resulting in robust and accurate expression quantification in HGSC cells. Both the QuantISH cancer cell classification and signal expression quantification on chromogenic images were evaluated through comparisons with visual evaluation in a tissue microarray sample set of HGSC. Furthermore, QuantISH expression quantification can also be performed on fluorescent RNA in situ hybridization (RNA-FISH) whole slide images as described in the Supplement.

Analysis of *CCNE1* expression in RNA-CISH images from HGSC patients showed that three groups based on *CCNE1* average expression show significantly different survival based on both PFI and OS. The predictive or prognostic value of the *CCNE1* expression varies in previous studies. Our results are in line with^41^, where *CCNE1* mRNA expression values were divided into three expression groups that also correlated to *CCNE1* amplification status (unamplified, gain, and amplification). Our result of non-significant survival association when patients were divided into two groups (high *vs*. low) at the median based on *CCNE1* expression are also corroborated with one study^25^. However, a study comparing strong expression to absent to moderate expression found no association with overall survival^24^ while we found there is a significant association to the overall survival in our results.

Our results highlight the importance of expression variability, as captured by the variability factor, and its potential as a biomarker using *DDIT3*, by demonstrating in two independent patient cohorts that heterogeneity in *DDIT3* expression predicts poor response, whereas the average expression is uninformative. Specifically, increased *DDIT3* heterogeneity is indicative of poor response regardless of the expression phenotypes of the individual cancer cells. Thus, our results suggest there are at least two types of biomarkers at the transcriptomics levels. The first category can be captured with the average expression whereas the second requires analysis of expression variability. For example, *CCNE1* features a constant level of variation between individual tumor cells in relation to the mean. Thus, for *CCNE1* the average expression is a clinically relevant marker and the *CCNE1* variability remains uninformative, whereas for *DDIT3* the opposite holds, which highlights the importance of accurately quantifying both properties.

While we have demonstrated utility of QuantISH, it has some limitations. First, QuantISH performance was evaluated using RNAScope data, which employs signal amplification prior to the imaging. Although QuantISH is technically applicable for analyzing images of other single molecule RNA-FISH techniques as well, the performance has not been assessed so far. Further, QuantISH is based on cell-specific intensity units, not on evaluating the exact number of signal dots. Thus, other tools, such as^13^, might be more applicable to single molecule RNA-FISH image quantification with very particular quantification needs. Second, very high target RNA abundance can cover nuclei borders or entire nuclei, which hinders the pipeline reconstructing the morphology of the underlying nuclei efficiently. This might lead deteriorated performance of the demultiplexing step. Third, the default cell segmentation module is based on CellProfiler that assumes that cells are mostly similar in size and feature a smooth morphology, which is suitable, for example, for carcinoma cell detection, but for cell types with more complex morphologies other specialized segmentation algorithms might be required. Fourth, the default cell type classification module uses low-pass filtering, which should be deactivated when working with images of highly complex tissue morphologies where the neighboring cells are of diverse types.

Taken together, our results show that QuantISH quantifies RNA expression levels and their variability in individual cells in a robust and accurate manner. While being the first pipeline that allows end-to-end quantitative analysis of RNA-CISH images, its modular design enables easy adaptability to different image sets and integration of novel computational methods, such as deep learning-based methods. As an open platform with thorough documentation, QuantISH paves the way to fully exploit the possibilities of RNA-ISH technology in future clinical applications.

## Supplementary information

Supplementary Material

## Data Availability

Whole slide images from the HERCULES/DECIDER cohort and tissue microarray images from the Helsinki cohort are available from the authors upon requests. QuantISH is openly available with source code and documentation at https://github.com/sanazjml/QuantISH_pipeline.
