# Supplementary material for: QuantISH: RNA in situ hybridization image analysis framework for quantifying cell type-specific target RNA expression and variability
Source: Lab Invest. 2022 Feb 15;102(7):753–61. doi: 10.1038/s41374-022-00743-5 (PMC9249626; doi:10.1038/s41374-022-00743-5)
Supplement: Supplementary file 1 — Supplementary Material [file 41374_2022_743_MOESM1_ESM.pdf]

Supplementary material

QuantISH: RNA *in situ* hybridization image analysis framework for quantifying cell type-specific target RNA expression and variability

Sanaz Jamalzadeh[1], Antti Häkkinen[1], Noora Andersson[1], Kaisa Huhtinen[2], Anna Laury[1], Sakari Hietanen[3], Johanna Hynninen[3], Jaana Oikkonen[1], Olli Carpén[1,2,4], Anni Virtanen[4,†], Sampsa Hautaniemi[1,†]

Supplementary methods

RNA *in situ* hybridization and imaging

Chromogenic RNA-ISH: We used freshly cut 3 μm sections of the TMAs and RNAscope 2.5 HD detection kit-BROWN (#322310, ACDBio, Milano, IT) for target mRNA detection. TMA sections were baked for 1 h at 56 °C, deparaffinized and treated with hydrogen peroxide for 10 min at room temperature. Target retrieval was performed for 15 min at 98 °C, followed by protease plus treatment for 15 min at 40 °C. Target probes Hs-DDIT3-C1 (#311131), Hs-CCNE1 (#470021), as well as the positive and negative control probes Hs-PPIB (#313901) and DapB (#310043), respectively, were hybridized for 2 h at 40 °C followed by signal amplification steps. The samples were incubated for 60 min with AMP 5 reagent. The sections were next treated with DAB for 10 min at room temperature followed by counterstaining with 50% hematoxylin. The sections were dipped in ammonium water and dehydrated before mounting. All TMAs were digitalized using 3DHISTECH Pannoramic 250 FLASH II digital slide scanner.

Fluorescent RNA-ISH: RNA *in situ* hybridization was performed on freshly cut 3 μm formalin-fixed paraffin embedded (FFPE) tissue sections using RNAScope Multiplex Fluorescent Reagent Kit Version 2 for target detection (#323100, Advanced Cell Diagnostics) according to the manual. In short, tissue sections were baked for 1 h at 60 °C, then deparaffinized and treated with hydrogen peroxide for 10 min at room temperature (RT). Target retrieval was performed for 15 min at 98 °C, followed by protease plus treatment for 15 min at 40 °C. All RNAScope probes (Table S1) were hybridized for 2 h at 40 °C followed by signal amplification, and developing of HRP channels was performed according to the manual. TSA Plus fluorophores fluorescein (1:750 dilution), Cyanine 3 (1:1500 dilution), and Cyanine 5 (1:3000 dilution) (NEL744001KT, Perkin Elmer) were used for signal detection. The sections were counterstained with DAPI, and mounted with ProLong Gold Antifade Mountant (P36930, Invitrogen). Images were generated using 3DHISTECH Pannoramic 250 FLASH II digital slide scanner at Genome Biology Unit supported by HiLIFE and the Faculty

of Medicine, University of Helsinki, and Biocenter Finland. All samples were scanned using 40x magnification with extended focus and 7 focus levels.

Table S1. The probe used in fluorescent RNA *in situ* imaging and its RNA scope catalog number.

**Probe name**	**Catalog number**
Hs-DDIT	311131

Quantitative analysis of whole slide RNA *in situ* hybridization images

Channel separation: We used CaseViewer (version 2.3.0) to read the MRXS immunofluorescence image, and to separate the different channels into the DAPI staining (nuclei), and fluorescein (FITC 38 HE), cyanine 3 (TRITC 48 HE), and cyanine 5 (Cy5) target RNA channels for gene expression quantification (Supplementary Figure 2).

Tiling the DAPI images: As the full whole slide images are too resource demanding for CellProfiler segmentation, the DAPI channel images were cropped into four tiles, each of which was segmented separately and assembled back into a whole slide segmentation for downstream analysis.

Cell segmentation: We used CellProfiler software (version 3.1.8) for segmenting the DAPI staining. The non-default parameters that were determined experimentally were as follows: typical diameter 18 to 56 pixels, thresholding using adaptive Otsu's method (i.e. local not global), clumped object detection and splitting using shape (i.e. not intensity), and low-resolution speedups were disabled. An example is shown in Supplementary Figure 5A.

Cell type classification: The segmented objects were classified into cancer, immune, and stromal cells using the DAPI staining and its segmentation. For this, we extracted the area, the mean nucleus stain intensity, and the eccentricity of each segmented object (Supplementary Figure 5B).

These features were selected based on the same logic as for the chromogenic images. Subsequently, we trained a supervised quadratic classifier using 402 cells with the properties mentioned above and the desired cell types (Supplementary Figure 5C). Afterwards, the cell types were predicted in untrained images (Supplementary Figure 5D). In order to improve the classification accuracy, the classification involved one artifact class in addition to the cancer, immune, and stromal classes, which was trained to represent the small segmentation artifacts in classification. The classifier was implemented in MATLAB and was trained with uniform class priors. The classification was filtered using spatial information from the neighboring cells, as described for the chromogenic images in the main manuscript.

RNA signal quantification: Cross-channel fluorescence bleed of Cy5, FITC, and TRITC staining was reduced by finding a suitable basis for the intensity data of all pixels near the principal axes (channels) using power iteration. Note that this is done at the pixel level, not at the cellular level. Since some RNA signals are localized in the cytoplasm of the cells, we also expanded the segmentation corresponding to the cancer, immune, and stromal nuclei to include the cellular cytoplasm, as described for the chromogenic images in the main manuscript. Next, the fluorescence intensity signal was quantified using the negative response of a Laplacian of Gaussian filter with standard deviation of unity. The value was tuned manually, and the kernel width roughly corresponds to the diameter of an observed RNA spot in our images. This procedure filters out background variations and cellular autofluorescence, leaving intensity blobs of the specified size.

Variability factor formulation

The weighted mean expression and its weighted variance for each TMA (whole slide) spot/patient are computed as follows:

$$m=\sum_i \left(\frac{Area_i}{\sum_i Area_i}\right) * \left(\frac{Intensity_i}{Area_i}\right) = \left(\frac{\sum_i Intensity_i}{\sum_i Area_i}\right)$$

$$v=\sum_i \left(\frac{Area_i}{\sum_i Area_i}\right) * \left(\frac{Intensity_i}{Area_i} - m\right)^2$$

where m is the mean expression in each spot/patient. $Area_i$ is the area of each individual cell, and the $Intensity_i$ represents the total (sum) intensity inside each cell. To compute the variability factor, we regressed out the mean from variability. First, we fit a linear regression model of expression on the raw variance in logarithmic spaces:

$$\log v \sim \alpha_1 \log m + \alpha_0$$

and obtained the estimated parameters α_1, α_0 of the linear regression. Following this, the expected log-variance due to the mean is:

$$\widehat{\log v} = \widehat{\alpha}_1 \log(m) + \widehat{\alpha}_0$$

which is then regressed out from the variability measurements:

$$\log v_{factor} := \log v - \widehat{\log v}$$

that is:

$$v_{factor} \sim v / m^{\widehat{\alpha}_1}$$

which is a generalization of the Fano factor ($\widehat{\alpha}_1 = 1$), and also of the coefficient of variation ($\widehat{\alpha}_1 = 2$), and the variance ($\widehat{\alpha}_1 = 0$), with arbitrary power-law relationship between the variance and the mean. Once the relationship has been configured, the variability factor can be computed for individual samples, just like the mean and the variance.

Supplementary Figures

Supplementary Figure 1. Pre-processing of the TMA images for downstream analysis

A. A linear transformation is used to align the frames from the slide scanner and to remove the overlapping areas in the MRXS image tiles. **B.** For tissue microarray images, the TMA spots are annotated and cropped from the whole slide image for downstream analysis.

Supplementary Figure 2. Pre-processing of the whole slide images for downstream analysis

Channel separation of immunofluorescence whole slide images to the DAPI, FITC, TRITC, and Cy5 channels.

Supplementary Figure 3. Overview of cell type classification features and training set for TMA analysis

A. Feature extraction for the cell classification, including area, mean-intensity, and eccentricity for each object inside TMA. **B.** Scatter plot of the training data in the feature space and a decision boundary plot including the different cell type classes.

Supplementary Figure 4. Selection of the reliable TMAs for survival analysis using positive control images

Otsu's thresholding method for two class classification of *PPIB* intensities resulted in separating low and high intensity groups in the *PPIB* intensity is used to select reliable images for quality control purposes.

Supplementary Figure 5. QuantISH image analysis pipeline steps from pre-processing to RNA quantification for whole slide images

A. Cell nucleus segmentation of the separated DAPI channel **B.** Extracting the segment features for training the cell type classifier. **C.** Decision boundary plot representing well-separated classifier features in the training data. **D.** Cell-type classification of a whole slide image.

Supplementary Figure 6. *DDIT3* average expression is uninformative of survival in HGSOC patients

DDIT3 cancer cell average expression is not associated with patients survival across the 92 HGSOC patients in this cohort. This in in contrast to the *CCNE1* average expression (cf. Figure 2C) or the *DDIT3* variability (cf. Figure 4C), both of which correlate with the platinum-free interval.

Supplementary Figure 7. *CCNE1* variability factor does not significantly change over three *CCNE1* expression groups

Variability factor does not significantly differ both between low and medium *CCNE1* expression (p=0.67) and medium and high *CCNE1* expression groups (p=0.95).

Figure S1

A.

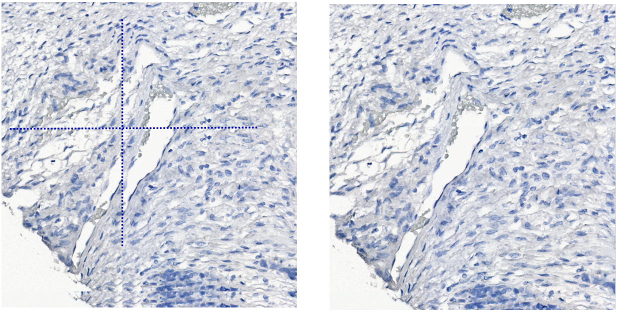

B.

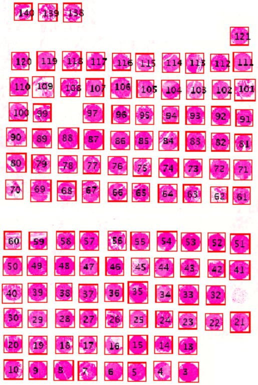

x	y	w	h
99584	326820	9984	9984
87040	326564	8704	9472
73984	327076	9216	9216
62208	326820	9216	9216
49152	326564	9472	9472
36864	326052	9728	9984
24320	326308	9216	8960
11776	325796	9472	9472
99584	314279	8960	8704
87552	313768	8704	8704
74240	313512	9472	9472
62464	314024	8960	8960
49408	313768	8960	8704
37632	313768	8704	8704
24320	313768	9216	8960
11776	313512	9216	8704
125440	301995	9728	9984
112896	302507	8704	8192
99584	301995	8960	8192
88064	301227	8448	8448
74752	300971	8960	8704
62720	300715	8704	8704
49664	300715	9216	8960
37632	300459	9472	9216

Figure S2

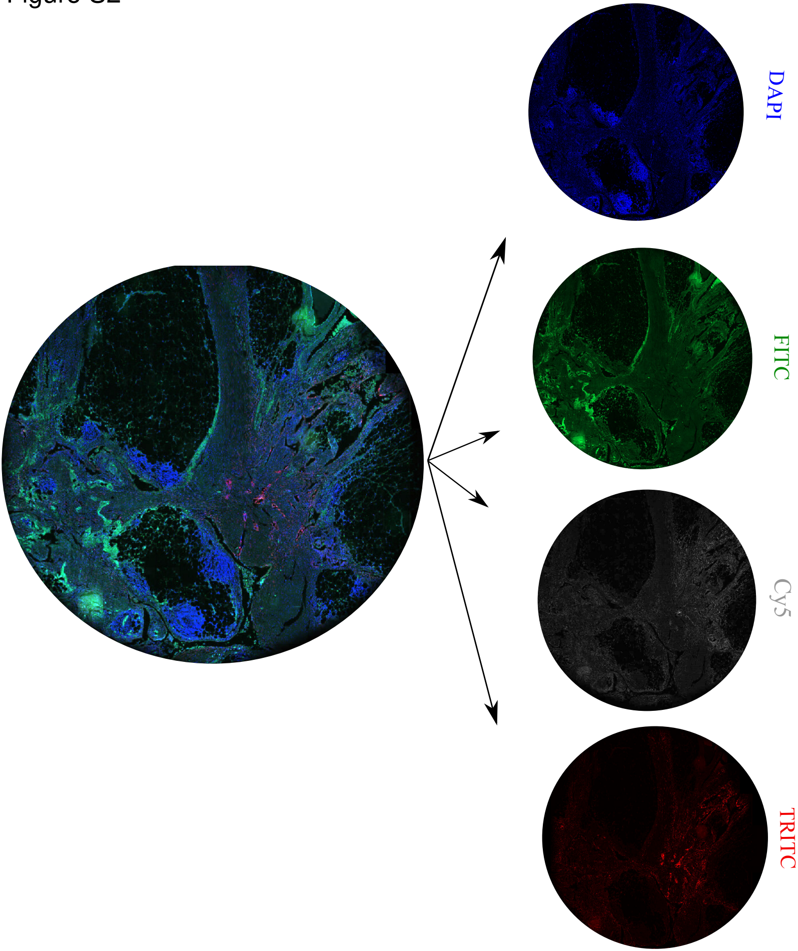

Figure S3

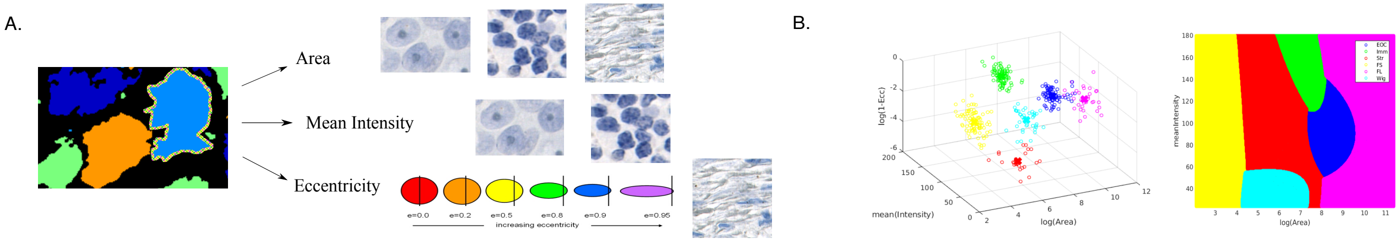
A.
Area
Mean Intensity
Eccentricity
e=0.0
e=0.2
e=0.5
e=0.8
e=0.9
e=0.95
increasing eccentricity
B.
log(1-Ecc)
mean(Intensity)
log(Area)
meanIntensity
log(Area)
EOC
Imm
Str
FS
FL
Wig

Figure S4

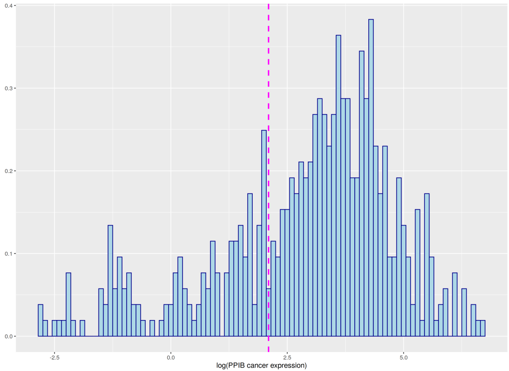

Figure S5

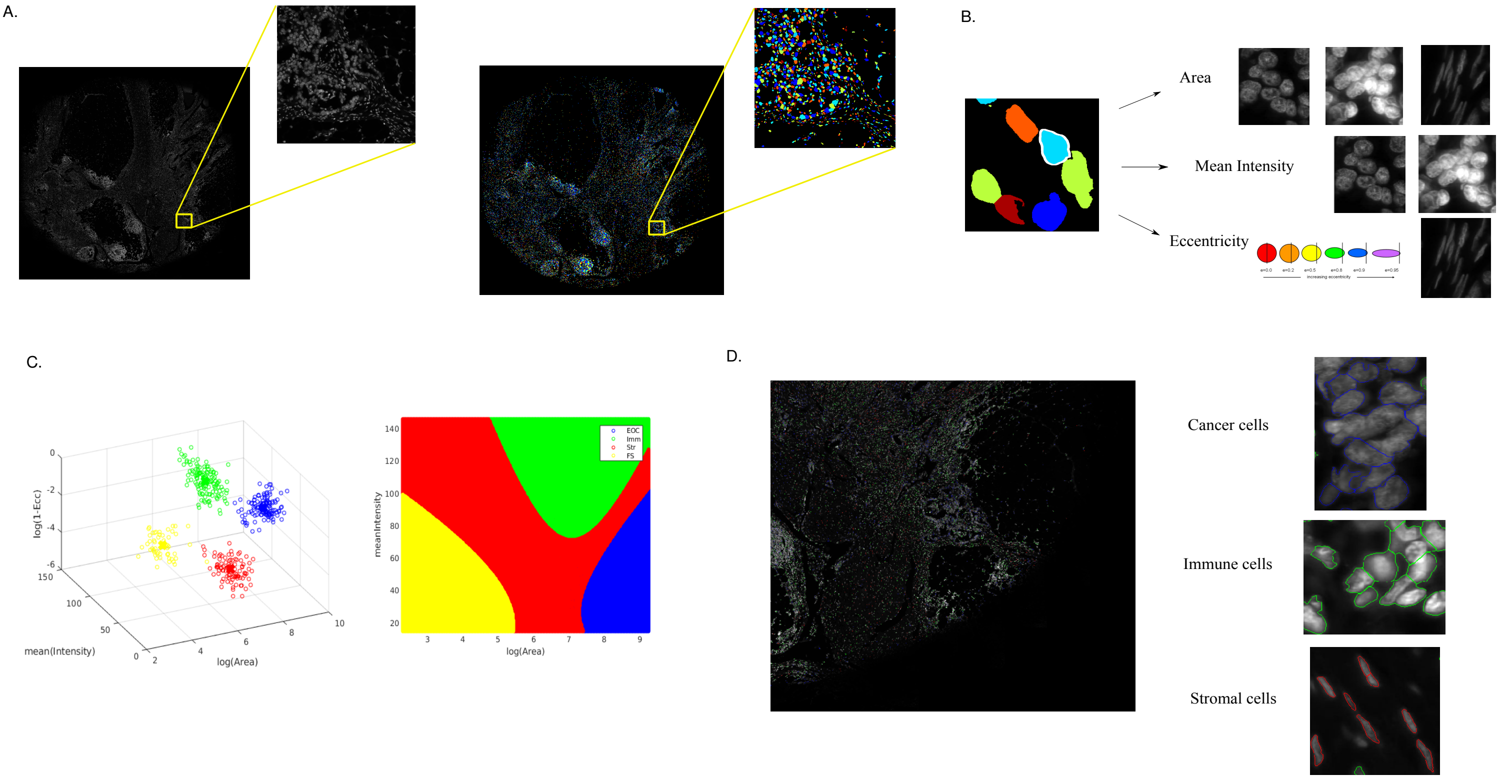
A.
B.
Area
Mean Intensity
Eccentricity
C.
log(1-Ecc)
mean(Intensity)
log(Area)
EOC
Imm
Str
FS
meanIntensity
log(Area)
D.
Cancer cells
Immune cells
Stromal cells

Figure S6

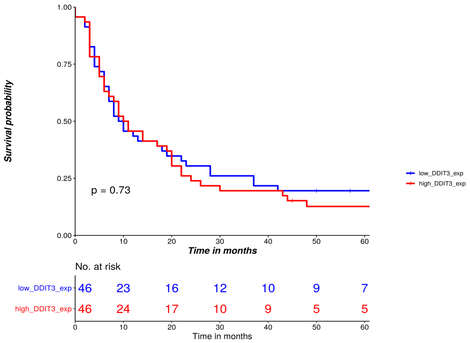

1.00
0.75
0.50
0.25
0.00
Survival probability
p = 0.73
0
10
20
30
40
50
60
Time in months
low_DDIT3_exp
high_DDIT3_exp
No. at risk
low_DDIT3_exp 46 23 16 12 10 9 7
high_DDIT3_exp 46 24 17 10 9 5 5
Time in months

Figure S7

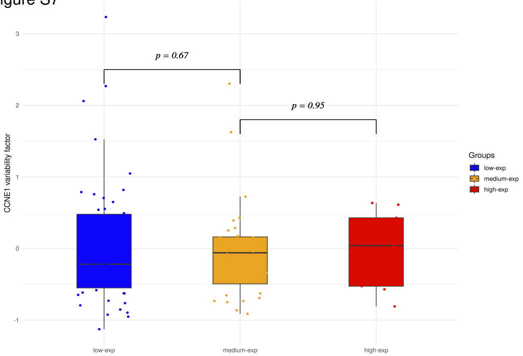

p = 0.67
p = 0.95
3
2
1
0
-1
CCNE1 variability factor
low-exp
medium-exp
high-exp
Groups
low-exp
medium-exp
high-exp
